# Pulmonary Hypertension Drives Prognosis in Idiopathic Pulmonary Fibrosis: Insights from the European IPF Registry

**DOI:** 10.3390/jcm14207352

**Published:** 2025-10-17

**Authors:** Andreas Guenther, Silke Tello, Marc Carre Schoppe, Joern Pons-Kuehnemann, Werner Seeger, Johannes Stiben, Khodr Tello, Maria Molina Molina, Carlo Vancheri, Bruno Crestani, Ekaterina Krauss

**Affiliations:** 1 European IPF/ILD Registry & Biobank (eurIPFreg/Bank, eurILDreg/Bank), 35392 Giessen, Germany; 2 Center for Interstitial and Rare Lung Diseases, Universities of Giessen and Marburg Lung Center (UGMLC), Justus-Liebig-University Giessen, Member of the German Center for Lung Research (DZL), Klinikstrasse 36, 35392 Giessen, Germany; 3 Agaplesion Lung Clinic, Evangelisches Krankenhaus Mittelhessen, Paul-Zipp Str. 171, 35398 Giessen, Germany; 4 Cardio-Pulmonary Institute (CPI) Klinikstr. 33, 35392 Giessen, Germany; 5 Institute for Lung Health (ILH), 35392 Giessen, Germany; 6 Institute of Medical Informatics, Justus-Liebig University of Giessen, 35392 Giessen, Germany; 7 ILD Unit, Respiratory Department, Biomedical Research Institute of Bellvitge (IDIBELL), University Hospital of Bellvitge-IDIBELL, 08907 Barcelona, Spain; 8 Regional Referral Center for Rare Lung Diseases, University Hospital Policlinico, Department of Clinical and Experimental Medicine, University of Catania, 95124 Catania, Italy; 9 Competence Center for Rare Pulmonary Diseases, Hopital Bichat, 75018 Paris, France

**Keywords:** idiopathic pulmonary fibrosis (IPF), European Registry for idiopathic pulmonary fibrosis (eurIPFreg), European Registry for interstitial lung diseases (eurILDreg)

## Abstract

**Background/Objectives**: In patients with idiopathic pulmonary fibrosis (IPF), a progressive disease characterized by lung tissue scarring, the impact of comorbidities is only partially understood. In particular, the prognostic implications of pulmonary hypertension (PH) are yet to be fully disclosed. **Methods**: To identify distinct IPF phenotypes on the basis of comorbidities and functional data, we performed cluster mixed data retrospective analysis, as well as recursive partitioning analysis on a dataset of 324 patients from the European IPF Registry (eurIPFreg); all patients were classified as IPF on the basis of established guidelines. Diagnosis of PH was based on echocardiographic and right heart catheter criteria as indicated in the 2022 ESC/ERS guidelines. **Results**: Two distinct clinical clusters with significant survival differences were identified (*p* < 0.001). Cluster 1, with fewer comorbidities, had a median survival of 4.41 years, whereas Cluster 2, with higher rates of arterial hypertension, diabetes mellitus, cardiovascular disease, PH, and dyslipidemia, showed a shorter median survival of 2.85 years. Multivariate Cox regression analysis confirmed PH as a significant predictor of reduced survival (HR 2.03). Recursive partitioning (RP) revealed that FVC was the strongest prognostic indicator: FVC below 50% predicted poor survival, and among patients with a FVC above 50%, the presence of PH indicated a significantly worse outcome. **Conclusions**: In this real-world IPF cohort, comorbidity cluster and RP analysis identified PH as the most relevant comorbidity. The findings suggest that PH may be more prevalent and impactful in IPF than previously recognized, with implications for clinical management.

## 1. Introduction

Longer life expectancy and improved healthcare access result in increased comorbidity rates, leading to complex disease burdens, polypharmacy, and prolonged hospitalizations, with over 70% of hospitalized adults affected by polymorbidity [1]. Idiopathic pulmonary fibrosis (IPF) is a progressive lung disease frequently complicated by comorbidities that impact prognosis and management. Although treatments like pirfenidone and nintedanib slow disease progression, predicting outcomes remains challenging due to the disease’s heterogeneity [2,3,4].

Common comorbidities in IPF include pulmonary hypertension (PH), cardiovascular diseases (CVD), arterial hypertension (AH), dyslipidemia, arrhythmias, and pulmonary embolism, the latter of which occurs up to three times more frequently than in non-IPF populations.

CVDs, specifically coronary heart disease, are frequently observed in patients with IPF [5]. This predisposes IPF patients to a higher risk of acute coronary syndrome and a greater prevalence of significant coronary heart disease, which can range from 3% to 68% and remains one of the leading causes of mortality in this population [6]. Alongside coronary heart disease, IPF patients may also experience other cardiovascular comorbidities, including AH, dyslipidemia, and cardiac arrhythmias [7,8]. Also, individuals with IPF who have impaired lung function and limited mobility may face a heightened risk of developing pulmonary embolism (PE). Data reports indicate that the incidence of PE in IPF patients is nearly three times higher compared to individuals without IPF [9].

Diabetes mellitus (DM) is a systemic metabolic disorder characterized by chronically elevated blood sugar levels, leading to complications in various organs and systems. Recent extensive research has highlighted DM as a significant comorbidity in IPF [10]. Gastroesophageal reflux disease (GERD) is commonly observed in patients with IPF, and there is a well-established hypothesis suggesting that microaspirations resulting from gastroesophageal acid refluxate contribute to lung tissue damage, pneumonitis, increased epithelial permeability, and subsequent fibrotic proliferation [11,12,13,14]. Sleep apnea is a sleep disorder characterized by interrupted breathing during sleep. Patients with IPF often experience disrupted sleep patterns, with a considerable portion of the night characterized by oxygen saturation below 90%; this might contribute to symptoms such as fatigue, nocturnal cough, excessive daytime sleepiness, and insomnia [15,16]. Depression and anxiety are further comorbidities with high prevalence in IPF, leading to emotional and psychological challenges that require appropriate management and support [17]. In addition to the impact on quality of life, mental health issues, specifically depression and anxiety, have been observed to have a significant influence on individuals with IPF, contributing negatively to the overall disease burden.

PH in IPF is defined by the 2022 ESC/ERS guidelines as a mean pulmonary arterial pressure (mean PAP) higher than 20 mm Hg, a pulmonary vascular resistance (PVR) >2 Wood Units, and a pulmonary arterial wedge pressure ≤15 mm Hg, confirmed via right heart catheterization (RHC), which is usually performed after echocardiographic assessment as initial diagnostic tool. As reiterated in recent guidelines, several echo parameters such as tricuspid regurgitation velocity (TRvel) >3.4 m/s or a tricuspid annular plane systolic excursion/pulmonary artery systolic pressure (TAPSE/sPAP) ratio <0.55 mm/mmHg are highly suggestive of PH [18]. Conducting RHC should be performed independently of diagnostic (suspected PAH or CTEPH; preparation for transplant) or therapeutic measures. In borderline cases, specialists may prefer close monitoring over immediate RHC to balance diagnostic accuracy with prudent risk management. PH in IPF often leads to a further reduction in diffusion capacity (DLco), oxygenation, and exercise tolerance, as well as a greater need for supplemental oxygen, more acute exacerbations, and a higher mortality risk [19,20,21]. Vasoactive drugs in this PH group have shown mixed results in the past; however, in the recent INCREASE trial, an improved exercise capacity in PH-IPF has been observed in response to inhaled treprostinil [22].

Many IPF patients have a history of smoking, which increases the risk of emphysema. This can lead to the development of a “combined pulmonary fibrosis and emphysema” (CPFE), masking the true extent of lung impairment by counterbalancing the effects of these two diseases on lung function [23,24].

Despite significant progress in understanding comorbidities in IPF, there are still unmet needs in comprehensively evaluating their interaction and impact on survival prediction. Therefore, further research is required to understand their contribution to outcomes, as improved insights into these relationships could lead to more targeted and effective treatment strategies for IPF patients.

## 2. Study Objectives

1. To perform cluster analysis on an IPF cohort using comorbidities, alongside variables such as lung function, age, sex, smoking status, antifibrotic use, and to evaluate the effectiveness of this analysis in predicting patient outcomes.
2. To identify high-risk variables through recursive partitioning (RP) analysis and evaluate their role in predicting survival and disease progression in IPF.

## 3. Materials and Methods

The European IPF Registry (eurIPFreg) and the European IPF Biobank (eurIPFbank) were established in November 2009 within the European IPF network [25]. The registry recently expanded to include other interstitial lung diseases (ILDs) and became the European Registry for Interstitial Lung Diseases (eurILDreg) [26]. Both registries are registered in clinical trial databases (eurIPFreg: ClinicalTrials.gov, NCT02951416; eurILDreg: DRKS 00028968).

For this study, data from 324 consecutive IPF patients diagnosed between November 2009 and March 2023 at the Giessen site were analyzed. Patient enrollment and baseline data were recorded at the time of registry inclusion, with diagnoses confirmed through multidisciplinary evaluation by chest physicians, pathologists, and radiologists based on recent ATS/ERS/JRS/ALAT guidelines. Patients routinely underwent echocardiography for PH screening and—as deemed necessary on clinical grounds—also RHC. Data were sourced from patient records and registry questionnaires, with comorbidities defined according to the latest guidelines. Uncertainties were resolved through physician notes and medication records.

### Statistics

The statistical analyses were performed using R 3.4 (www.r-project.org). Descriptive statistics were used to summarize baseline characteristics of the study cohort. Continuous variables (e.g., age, BMI, and lung function) were reported as mean ± standard deviation (SD), and categorical variables (e.g., sex, smoking status, comorbidities) as counts and percentages. For lung function (FVC, DLCO), GAP stage, and 6-Min Walk Distance (6MWD), the measurement closest to baseline was used (allowing a margin of one adjacent record before or after registry entry). Data from post–lung transplant visits were excluded.

**Figure 1:**
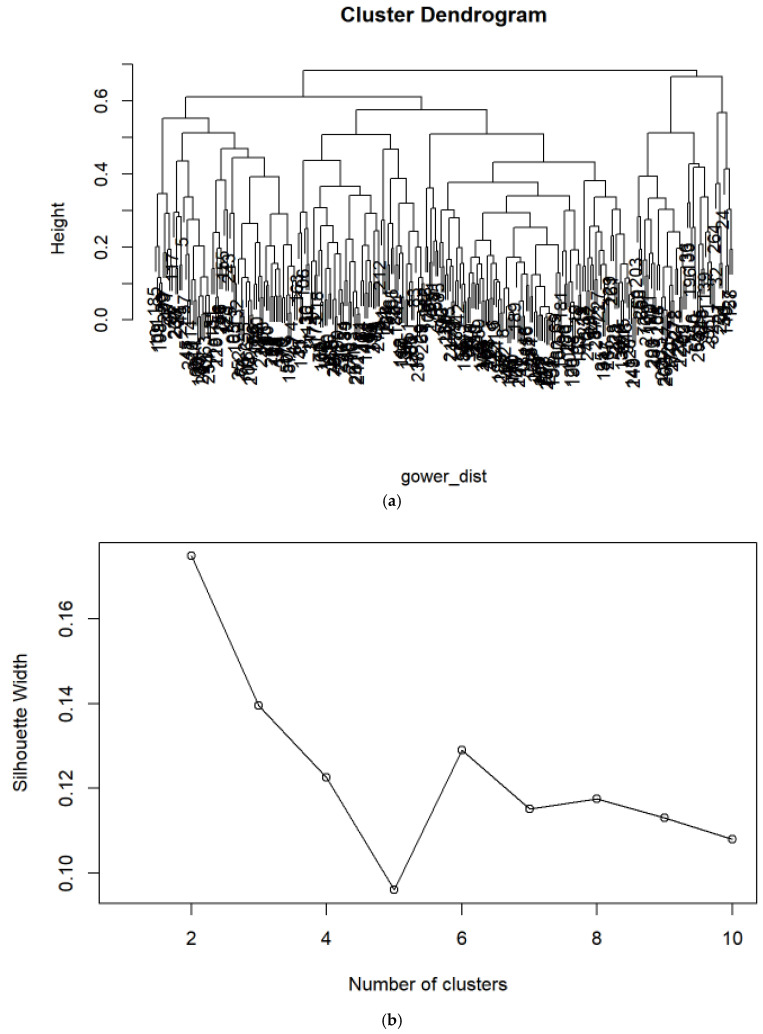
(**a**) Hierarchical Cluster Analysis of Comorbidities (Gower dissimilarity metric). The cluster dendrogram shows data points grouped by similarity, with connecting lines indicating distances and branches representing cluster mergers. (**b**) Silhouette width determined the optimal number of clusters as two.

Cluster analysis was applied to identify phenotypic subgroups based on comorbidities and demographic/functional variables (age, sex, smoking status, antifibrotic therapy, lung function). Because the dataset included both categorical and continuous variables, the Gower dissimilarity metric was used (Figure 1a). Hierarchical clustering (*hclust*) and Partitioning Around Medoids (PAM) were performed, with cluster quality evaluated by silhouette width, determining the optimal number of clusters as two (Figure 1b). Clusters were subsequently characterized using descriptive statistics and Kaplan–Meier survival curves.

Recursive partitioning (RP) with conditional inference trees (*ctree* package) was used to explore prognostic determinants of survival. This method recursively splits the dataset into more homogeneous subgroups according to independent variables. Stopping rules were applied based on minimum subgroup size and tree complexity. Both univariable and multivariable RP analyses were performed; the latter incorporated covariates and applied Bonferroni correction to control for multiple testing.

Survival analysis was conducted using Cox proportional hazards models, with results reported as hazard ratios (HRs) and 95% confidence intervals (CI). Univariable models assessed crude associations, while multivariable models were adjusted for key confounders (age, sex, BMI, and FVC group). Survival was visualized with Kaplan–Meier curves and risk tables, with significance set at *p* < 0.05. All analyses were based on individual patient-level variables rather than clustering, ensuring robustness of the prognostic conclusions. To address the 10 events-per-variable (EPV) principle, the number of covariates in the final models was restricted relative to the number of events. In line with a specific modeling strategy, only clinically relevant and statistically supported variables were included, and model stability was confirmed by comparison with reduced models, which produced consistent results.

Missing covariate data were addressed by imputation, generating several datasets analyzed separately, with pooled estimates reported. Because not all variables were available for all patients, sample sizes varied between analyses (e.g., cluster analysis: 287 patients; RP analysis: 324 patients with FVC and PH data; echocardiography subset: 266 patients).

This approach allowed the inclusion of as many patients as possible and reduced bias. The combined study approach—descriptive statistics, cluster analysis, recursive partitioning, and Cox regression—enabled both exploratory identification of comorbidity clusters and hypothesis-driven assessment of PH as an independent prognostic factor.

## 4. Results

The descriptive characteristics of the cohort are summarized in Table 1. Out of 342 patients, 266 (82.1%) underwent baseline echocardiography, with 72 (27.1%) showing signs of high probability of PH (SPAP > 46 mmHg, TRvel ≥ 3.4), and 194 (72.9%) showing no right heart strain (SPAP ≤ 46 mmHg, TRvel < 3.4). A total of 78 patients underwent RHC. Among these, PH (mPAP > 20 mmHg, PVR > 2 WU) was confirmed in 46 patients, representing 14.2% of the total cohort and 54.76% of the PH cohort. The remaining 45.24% of our PH cohort showed high-probability PH on echocardiography, but either were not primarily suggested to undergo RHC due to individual risk/benefit evaluations or opted against RHC. In the subjects undergoing RHC, the mean pulmonary capillary wedge pressure (PCWP) was found to be 6.54 ± 4.11 mmHg. A total of 8 patients (9.52% of the PH cohort) had combined pulmonary fibrosis and emphysema (CPFE).

Table 2 summarizes cluster analysis data, presenting variables categorized by two clusters, along with statistical tests such as Pearson and Wilcoxon. The variables are presented separately for Cluster 1 and Cluster 2, with corresponding *p*-values indicating the statistical significance of differences between the groups. Categorical variables are expressed as percentages with absolute counts (n), and continuous variables (age, BMI) are reported as mean ± SD.

### 4.1. Cluster Analysis of Comorbidities

Cluster 1 exhibited significantly lower rates of AH, DM, CVD, PH, and dyslipidemia than Cluster 2 (*p* < 0.001 for all), with Cluster 2 also having a significantly higher mean age (70.15 ± 8.72 vs. 66.35 ± 9.70 years, *p* = 0.0032), greater prevalence of arrhythmia (*p* = 0.0221), and a higher proportion of males (86.5% vs. 75.8%, *p* = 0.0381). No significant differences were found between clusters regarding CPFE, FVC (*p* = 0.17), familial IPF, GERD, lung malignancy, smoking, sleep apnea, depression, BMI, thyroid disease, neurologic-ischemic conditions, PE or other malignancies (*p* > 0.05 for all). Overall, Cluster 2 represented an older population with more cardiovascular comorbidities.

**Figure 2:**
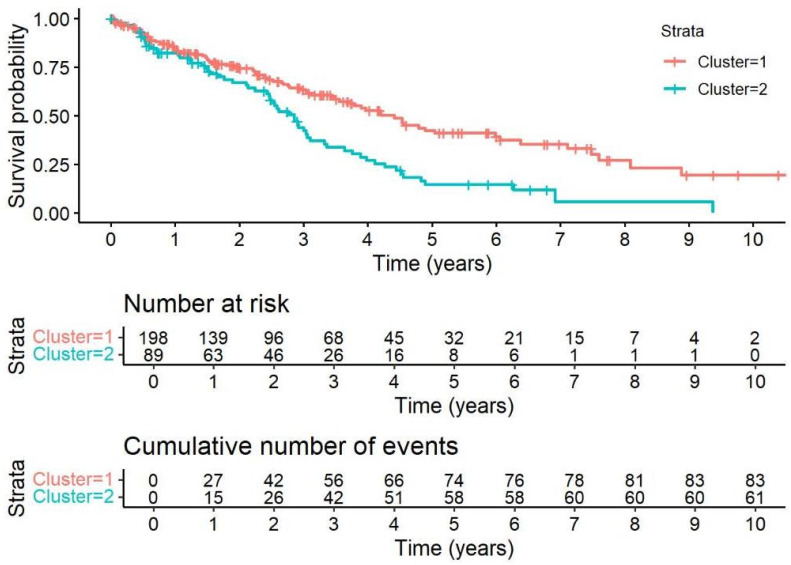
Survival Analysis of Cluster 1 and Cluster 2: Kaplan–Meier curves depicting number at risk and cumulative event (death) rates. Cluster 2 (n = 89) was associated with a 1.85-fold higher risk of death compared to Cluster 1 (n = 198), HR = 1.85 (95% CI: 1.33–2.58), *p* < 0.001; this association remained consistent in multivariate analysis.

Figure 2 illustrates survival differences between the two clusters, with Cluster 2 (n = 89) showing a 1.85 times higher hazard ratio (HR) for death compared to Cluster 1 (n = 198), with a 95% confidence interval of 1.325 to 2.582 (*p* < 0.001), which remained consistent in the multivariate analysis. Cox regression analysis in 324 patients (Table 3) identified PH as a significant predictor of poorer survival, with PH patients having a 2.8-fold higher risk of death (HR: 2.80 univariate, 2.03 multivariate, both *p* < 0.001). Higher baseline FVC was associated with lower mortality risk, with HR decreasing as FVC improved (*p* < 0.001). Age and BMI showed modest associations with survival, but these became non-significant in multivariate analysis (age HR: 1.02, *p* = 0.030 univariate; BMI HR: 0.96, *p* = 0.045 univariate). Other comorbidities like AH (*p* = 0.053 univariate), CVD (*p* = 0.062 univariate), and sex (*p* = 0.053 univariate) showed weak associations with survival but lacked statistical significance in multivariate analysis, while smoking and DM were not significant predictors.

### 4.2. Recursive Partitioning-Analysis

**Figure 3:**
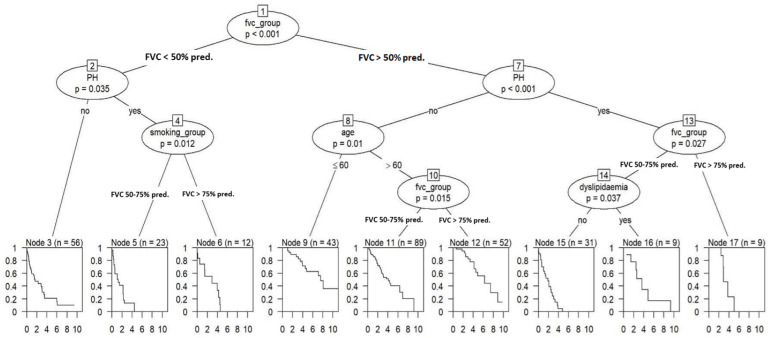
Regression tree analysis (ctree, univariate model). FVC Groups: 1—<50% predicted value, 2—50–75% predicted value, 3—>75% predicted value. Abbreviations: FVC% pred.—Forced Vital Capacity as a Percentage of Predicted Value; PH—Pulmonary Hypertension.

Figure 3 illustrates the results of RP using a “ctree” model on 324 IPF patients, where iterative data segmentation based on independent variables optimizes survival prediction. This is a type of decision tree algorithm that uses conditional inference procedures for binary, categorical, and continuous outcome variables. In our analysis, through five iterative steps, the RP method progressively divided the dataset into subsets (split nodes), resembling the growth of a decision tree, with the overarching goal of optimizing its complexity to enhance predictive performance. In the context of this analysis, the first and most pivotal determinant of patient outcome is the FVC value. Patients with an FVC of less than 50% of the predicted value serve as the initial focus of iteration. Within this subgroup, the subsequent outcome depends on the presence of PH as a comorbidity (*p* = 0.035), with smoking status further affecting prognosis (*p* = 0.012).

In subsequent iterations, attention shifts to patients falling into FVC categories 2 (50–75% predicted value) or 3 (>75% predicted value). Again, the direction of the iteration is substantially influenced by the presence of PH, a statistically significant factor (*p* < 0.001). If PH is absent, the iteration trajectory is markedly impacted by the patient’s age (over or under 60, *p* = 0.01). For patients aged over 60, further iteration steps once again exhibit a direct dependency on FVC values. Conversely, in cases where PH is present, survival outcomes are again primarily dependent on the FVC categories and then, in the FVC category >75%, on the presence of dyslipidemia (*p* = 0.037).

**Figure 4:**
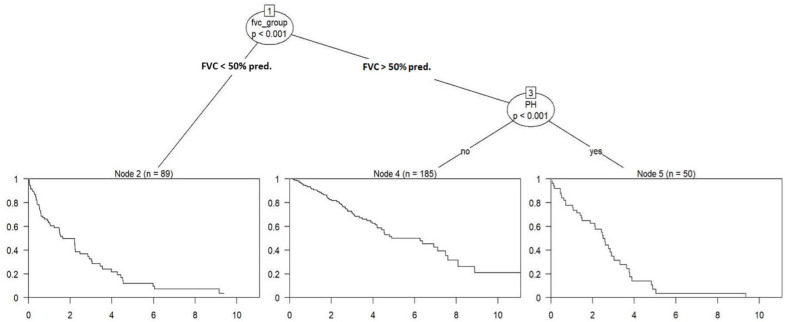
Regression tree analysis (ctree, multivariate model, adjusted after Bonferroni). FVC Groups: 1—<50% predicted value; 2—50–75% predicted value; 3—>75% predicted value. Abbreviations: FVC% pred.—Forced Vital Capacity as a Percentage of Predicted Value; PH—Pulmonary Hypertension.

**Figure 5:**
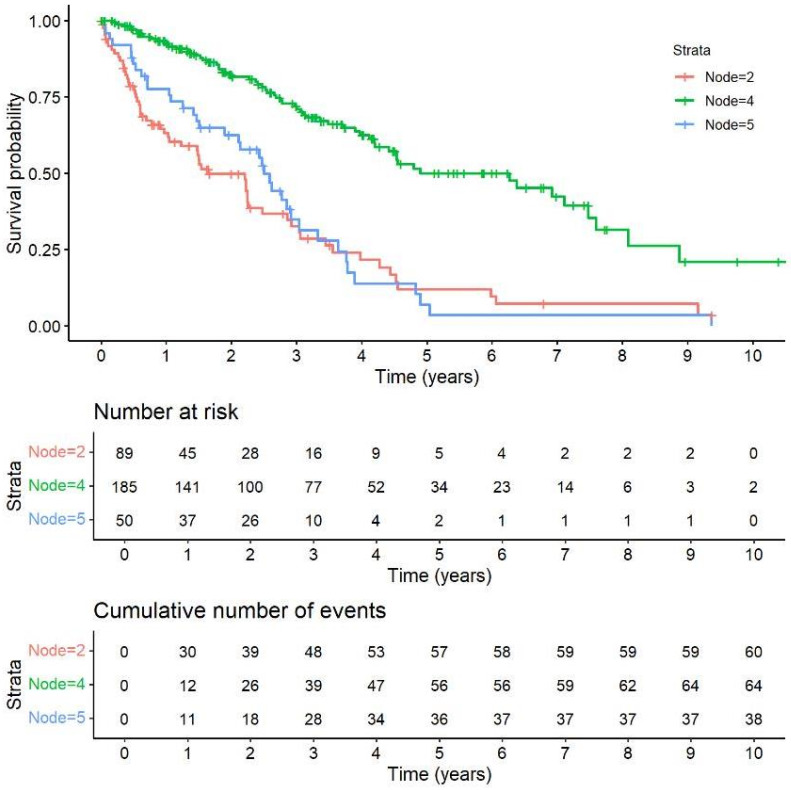
Survival analysis between the groups in the conditional inference tree (ctree) model.

In the subsequent multivariate analysis, presented in Figure 4, with rigorous data adjustment following Bonferroni correction, the initial pivotal outcome variable is the degree of restriction in lung function, as indicated by FVC. Patients falling into FVC category 1, characterized by FVC values < 50% of the predicted value, constitute node 2 (n = 89), representing 27.5% of the total cohort (Table 4). Notably, the HR is implicitly set at 1.00 in both univariable and multivariable analyses for node 2, given its function as the reference group in the analysis. The presence of PH plays a significant prognostic role, with a strong statistical association (*p* < 0.001) in patients with FVC categories 2 (50–75% predicted) and 3 (>75% predicted). Patients without PH, forming node 4 (185 observations, 57.1% of the cohort), show a substantially lower HR = 0.29 (95% CI: 0.20–0.41, *p* < 0.001) compared to the reference group (node 2, FVC <50%). In contrast, patients in node 5 (50 observations, 15.4% of the cohort), who show similar FVC values but the presence of PH, exhibit no significant survival difference as compared to the reference group (HR = 0.88, 95% CI: 0.59–1.33, *p* = 0.557), as presented in Table 5 and Figure 5.

## 5. Discussion

In this study, we identified two distinct IPF clusters with varying characteristics and outcomes, highlighting the critical role of relevant functional values and comorbidities, especially PH, in IPF risk assessment and treatment decisions. Cluster 2 exhibits higher rates of various comorbidities and an older age compared to Cluster 1 and represents the group with elevated cardiovascular risk factors and a 1.85-times higher risk of death. 

The ctree analysis identified FVC as the primary determinant of survival in IPF, serving as the initial and most influential split in the RP model. Patients with severely impaired lung function formed the highest-risk subgroup, in which PH further stratified prognosis, highlighting its additive role in patients with advanced physiological restriction. Within this context, smoking history contributed additional prognostic separation, reflecting the cumulative effect of exposure-related and vascular comorbidities. In patients with preserved or moderately reduced FVC, PH again emerged as a critical factor shaping survival trajectories. Among those without PH, age became a relevant discriminator. Conversely, in patients with coexisting PH and better-preserved lung function, the presence of dyslipidemia was associated with further risk differentiation, suggesting a synergistic effect between pulmonary vascular disease and systemic metabolic burden. This analysis revealed a hierarchical and multidimensional interaction between functional impairment, vascular comorbidity, and patient-related characteristics such as age, metabolic profile, and smoking history. The recurrent appearance of PH as a key branching determinant across FVC strata underscores its prognostic relevance, independent of baseline lung function, and supports the clinical utility of early cardiovascular evaluation in IPF, providing a framework for risk stratification based on distinct and clinically accessible phenotypes.

As mentioned, PH emerged as a significant predictor of poor survival, independent of the applied statistical procedure. In IPF patients, PH knowingly results in a further impairment of gas exchange and lung function through pulmonary vascular remodeling, increased vascular resistance and right ventricular dysfunction. Kimura et al. analyzed data from 101 consecutive IPF patients who underwent RHC during the initial work-up, although the reasons for performing RHC were not disclosed, and 76 patients not undergoing RHC were excluded. Using the Cox proportional hazards model, the study identified higher mean PAP and lower FVC at baseline as significant predictors of five-year survival, with a mean PAP greater than 20mmHg serving as a critical threshold for identifying higher-risk patients [27]. Lettieri et al. showed in a cohort of IPF subjects undergoing evaluation for lung transplantation that PH diagnosed by RHC correlated with mortality [28]. Hence, only the study of Kimura et al. addressed PH in a comparable patient cohort as we did, but their study only included patients undergoing RHC.

Age showed a modest, statistically significant association with survival, with higher age linked to a slightly higher HR in univariate analysis, while higher BMI was associated with a slightly lower HR, though this was not significant in multivariate analysis. Other comorbidities, such as AH, CVD, and sex, had varying associations with survival, but many were not statistically significant, suggesting that, while these factors may complicate IPF management, they do not directly impact progression, with GERD potentially worsening respiratory symptoms and lung function [11,12,13,14].

No significant difference in CPFE prevalence was observed across the clusters in our study. However, when diagnosed, CPFE requires tailored management addressing both fibrotic and emphysematous components, with smoking cessation being crucial due to the strong association between emphysema and smoking [29].

Also, no significant differences were found in the prevalence of malignancies, depression, anxiety, neurovascular, or thyroid diseases across IPF clusters, but regular screenings and addressing mental health concerns are important for timely detection and improving patient well-being.

Comparing our findings to previous cluster analyses in IPF or ILD is difficult due to variations in cohort size, methodology, statistical analysis, and the inclusion of diverse ILD subtypes, clinical characteristics, and treatment factors.

In the study conducted by Kreuter et al., the impact of comorbidities was investigated in 171 IPF patients, employing a multivariate Cox proportional HR model, adopting a comorbidome approach [30]. Further, TORVAN, a clinical prediction model similar to GAP score, has been developed by Torrisi et al. and validated for all-cause mortality in IPF, incorporating comorbidities as variables and enhancing the accuracy of survival prediction beyond physiological and functional data. In this study, some comorbidities showed a significant impact on survival prediction [21]. Similarly, Hyldgaard et al. conducted a study investigating the impact of comorbidities on the survival of IPF patients, and their findings align with some of our results regarding the prevalence of certain comorbidities [31]. Specifically, CVD, depression, AH, and DM were identified as the most commonly observed comorbidities in both studies. This consistency in results further strengthens the significance of these comorbid conditions in influencing the prognosis and outcomes in IPF.

Bordas-Martínez et al. conducted a cluster analysis of 136 IPF patients, identifying three distinct clusters based on disease behavior. Cluster 1 was characterized by a delay of over two years in starting antifibrotic treatment, a history of smoking, emphysema, and a consistent UIP pattern on imaging, with a higher prevalence of hiatal hernia and CPFE. Cluster 2 had longer survival, a longer duration of antifibrotic treatment, and a lower rate of disease progression, with earlier diagnosis and a shorter time from symptom onset to treatment, including some patients identified through familial screening. Cluster 3 was associated with rapid disease progression, metabolic syndrome, cardiovascular comorbidities, obesity, and a higher prevalence of sleep apnea. PH was suspected in 32% of patients on echocardiography and diagnosed in 6% by RHC, with the prevalence of PH being similar across all three clusters [32].

Kam et al. performed a hierarchical cluster analysis in an ILD cohort, identifying four distinct ILD phenotypes with varying characteristics, and incorporating additional variables such as asthma, comorbidities count, imaging patterns, and tuberculosis history, while also examining the prevalence of PH across the phenotypes, though no significant differences were found (*p* = 0.946) [33].

Aoshima et al. conducted a hierarchical cluster analysis on 337 patients with idiopathic interstitial pneumonias to explore the associations of clinical phenotypes with acute exacerbation and survival, considering factors such as dust exposure, biomarkers, and treatment data. PH was not addressed in the study [34].

Adegunsoye et al. identified distinct ILD phenotypic clusters by examining baseline characteristics, including demographics, laboratory values, imaging patterns, environmental exposures, and comorbidities like emphysema, hypothyroidism, and GERD, as well as values of the diameter of the pulmonary artery (but not PH diagnosis); though many of these variables were center-specific and of limited relevance compared to our findings [35].

Prior et al. conducted a cluster analysis of 150 IPF patients, identifying distinct patterns of comorbidities across four clusters; with Cluster 1 having fewer comorbidities, Cluster 2 showing a higher prevalence of ischemic heart disease, Cluster 3 featuring more emphysema and airway obstruction, and Cluster 4 displaying a higher incidence of anxiety, depression, pain disorders, and a greater overall comorbidity burden (PH, pulmonary embolism, and malignancies were not evaluated in the study) [36].

Although the mentioned studies exhibit variations in study concepts, statistical methodologies, cluster sizes, and performance of multivariate analysis (if any), the key distinction lies in the center-specific emphasis on the relevance of comorbidities. Each research group incorporates factors and comorbidities that they believe are of particular importance to the respective centers.

In our study, we analyzed a comprehensive set of comorbidities that appeared to be of particular importance in this context, i.e., PH, malignant diseases, neurovascular (ischemic) disorders, cardiac arrhythmia, etc. The routine clinical assessment for PH, driven by the overall focus on PH in Giessen, allowed us to shed light on its significance alongside other comorbidities. Further novelty is the usage of RP in the outcome analysis of our IPF cohort.

Our study has several strengths, including a comprehensive dataset with a large sample size and patients distributed across each cluster, which establishes a robust foundation for the drawn conclusions. In addition, the Bonferroni correction applied during multivariate analysis further ensures that our findings are statistically robust, particularly in the context of multiple testing, thus minimizing the risk of false-positive results. Overall, the methodology applied in this study, including the use of imputation for missing data and the selection of appropriate patient subsets for specific analyses, ensures that the results are as robust and representative as possible, despite the inherent challenges of working with real-world clinical data. While there might be some limitations in terms of generalizability in IPF as such, the approaches used allow for the exploration of important clinical patterns in the patient population and offer insights into risk stratification and survival in IPF patients.

The identified clusters exhibit distinct patterns and characteristics, demonstrating the effectiveness of the clustering algorithm in grouping patients based on similarities and revealing meaningful subgroups within the dataset. From our analysis, including RP, several important insights emerge. In line with previous reports, FVC is a crucial determinant of patient outcome [37]. The presence of PH is the second key determinant of further branching in the iteration process and determines IPF prognosis.

While the partial absence of RHC for PH diagnosis in some patients may be viewed as a limitation, it reflects the clinical reality. RHC in Group 3 PH patients is typically reserved for cases with a strong echocardiographic probability of PH, and diagnostic or therapeutic consequences, as outlined in recent guidelines [18]. In borderline cases, specialists often opt for close monitoring. This approach balances diagnostic accuracy with patient safety, given the invasive nature and risks of RHC [38]. John et al. showed that in patients with echocardiographic high-probability PH, but normal or mildly elevated mean PAP at rest, the TAPSE/sPAP ratio independently predicts clinical worsening and correlates strongly with exercise capacity and hemodynamics [39]. Despite this limitation, our multivariate Bonferroni analysis identified PH as a major prognostic factor. This approach mirrors real-world clinical practice and highlights the importance of diagnostic strategies in understanding PH’s impact on patient outcomes.

Furthermore, hierarchical clustering was used as an exploratory tool to visualize comorbidity patterns, while the main prognostic analyses were based on individual patient-level variables and Cox regression, ensuring robustness of conclusions. Finally, although rare, myeloproliferative neoplasms represent a recognized cause of Group 5 PH and can overlap with fibrotic lung disease phenotypes; however, no patients in our cohort were formally diagnosed with these conditions [40].

As for the varying number of patients across different analyses, while this selective inclusion of patient subgroups can complicate the generalization of results, it is a fact in real-world data analysis where missing values are unavoidable and certain variables might not be consistently available for all patients. The use of imputation models in dealing with missing data, together with multivariate Cox regression models adjusted for confounding variables, helps to mitigate the impact of these inconsistencies by controlling for factors that might otherwise skew the results.

Our observations in this study gain particular relevance in the context of the recent TETON trial, which investigated inhaled treprostinil in IPF patients, illustrating the therapeutic momentum in targeting pulmonary vascular pathways in fibrotic lung disease [41]. Our findings suggest that identifying and characterizing IPF patients with PH—especially those with preserved lung function but vascular involvement—may be critical for refining trial populations and for guiding clinical decision-making in the era of emerging antifibrotic and vasodilatory strategies.

Our study is relevant for several reasons. It contributes to a better understanding of the heterogeneity of IPF and the complex interplay between comorbidities and patient characteristics in determining disease outcomes. By identifying specific patient subgroups with distinct prognostic profiles, the study provides valuable insights that can help to set up personalized treatment strategies and improve patient management. Additionally, the use of advanced statistical techniques such as cluster analysis and RP adds depth to the analysis, allowing for a more nuanced exploration of the data and a comprehensive assessment of factors influencing survival in IPF.

## 6. Conclusions

In summary, cluster analysis and RP undertaken in our real-world IPF cohort highlight the important prognostic role of PH in IPF and underscore its importance of comorbidities in IPF management. Our study provides strong evidence that rigorous screening for PH should be part of the initial work-up for IPF, as treatment options for PH-IPF now exist and have been authorized at least in the US.

## Figures and Tables

**Table 1:** Descriptive variables of the study cohort at baseline.

Variables	The Data Presented as % of the Study Cohort
Age (groups)	(n = 324)
18–59	20.37
60 + 75	58.95
≥ 76	20.68
Men	78.40
Women	21.60
Antifibrotics (pirfenidone, nintedanib)	80.56
BMI (groups)	(n = 319)
≤18.4	0.32
18.5–29.9	68.65
≥30	31.03
Smoking status (groups)	(n = 298)
Active smoker	3.02
Ex-smoker	72.48
Never smoked	24.50
Pack years (mean value ± SD)	27.4 ± 19.0
Long-term oxygen therapy	43.94
6MWD (groups)	(n = 192)
≥350 m	65.10
<350 m	34.90
NYHA (groups)	(n = 254)
NYHA I	19.69
NYHA II–III	75.59
NYHA IV	4.72
Familial IPF	19.77
GAP stage (groups)	(n = 227)
I	48.46
II	42.73
III	8.81
Death	44.14
Lung transplant	5.86

Abbreviations: BMI—Body Mass Index; 6MWD—Six-Minute Walk Distance; NYHA—New York Heart Association; IPF—Idiopathic Pulmonary Fibrosis; IPAF—Interstitial Pneumonia with Autoimmune Features; GAP—Gender, Age, and Physiology Score; n—Number; SD—Standard Deviation.

**Table 2:** Disparities between the IPF clusters at baseline.

Variable	Cluster 1 (n = 198)	Cluster 2 (n = 89)	*p*-Value
AH	24.8% (49/198)	87.6% (78/89)	**<0.0011**
DM	15.7% (31/198)	38.2% (34/89)	**<0.0011**
PH	10.1% (20/198)	61.8% (55/89)	**<0.0011**
Dyslipidemia	11.1% (22/198)	30.3% (27/89)	**<0.0011**
CVD	18.2% (36/198)	77.5% (69/89)	**<0.0011**
Age (mean ± SD)	66.35 ± 9.70	70.15 ± 8.72	0.0032
Arrhythmia	11.1% (22/198)	21.4% (19/89)	0.0221
Sex_group (male)	75.8% (150/198)	86.5% (77/89)	0.0381
CPFE	3.54% (7/198)	7.87% (7/89)	0.1151
FVC_group			0.1741
1. <50% pred.	1: 27.8% (55/198)	1: 27.0% (24/89)	
2. 50–75% pred.	2: 45.0% (89/198)	2: 55.1% (49/89)	
3. >75% pred.	3: 27.3% (54/198)	3: 18.0% (16/89)	
Familial_IPF	18.2% (36/198)	12.4% (11/89)	0.2181
GERD	8.59% (17/198)	5.62% (5/89)	0.3821
Lung_malignancy	4.55% (9/198)	6.74% (6/89)	0.4391
Smoking_group			0.5061
1. Current smoker	1: 3.54% (7/198)	1: 1.12% (1/89)	
2. Former.smoker	2: 71.21% (141/198)	2: 74.16% (66/89)	
3. Never smoked	3: 25.25% (50/198)	3: 24.72% (22/89)	
Sleep_apnea	13.1% (26/198)	15.7% (14/89)	0.5571
Antifibrotics	80.3% (159/198)	83.2% (74/89)	0.5691
Depression_anxiety	6.06% (12/198)	7.87% (7/89)	0.571
BMI (mean ± SD)	28.0 ± 4.29	27.6 ± 4.49	0.622
Other_malignancies	13.1% (26/198)	11.2% (10/89)	0.6541
Thyroid_disease	20.2% (40/198)	18.0% (16/89)	0.661
Neurological_ischemic	7.07% (14/198)	7.87% (7/89)	0.8111
PE	5.56% (11/198)	5.62% (5/89)	0.9831

Abbreviations: AH—Arterial Hypertension; BMI—Body Mass Index; CPFE—Combined Pulmonary Fibrosis and Emphysema; CVD—Cardiovascular Diseases; DM—Diabetes mellitus; FVC% pred.—Forced Vital Capacity as a Percentage of Predicted Value; GERD—Gastroesophageal Reflux Disease; IPF—Idiopathic Pulmonary Fibrosis; PH—Pulmonary Hypertension; PE—Pulmonary Embolism; SD—Standard Deviation; n—Number.

**Table 3:** Impact of Comorbidities and FVC on Survival (Cox Regression, time_to_event_years).

Variable	HR (Univariate)	HR (Multivariate)
PH	2.80 (2.04–3.86, ***p* < 0.001**)	2.03 (1.42–2.90, ***p* < 0.001**)
FVC Group		
<50% pred.	-	-
50–75% pred.	0.44 (0.31–0.62, ***p* < 0.001**)	0.51 (0.35–0.73, ***p* < 0.001**)
>75% pred.	0.19 (0.12–0.32, ***p* < 0.001**)	0.23 (0.14–0.40, ***p* < 0.001**)
Age	1.02 (1.00–1.03, ***p* = 0.030**)	1.01 (0.99–1.03, *p* = 0.235)
BMI	0.96 (0.93–1.00, ***p* = 0.045**)	0.98 (0.94–1.02, *p* = 0.244)
AH	1.36 (1.00–1.85, *p* = 0.053)	1.21 (0.84–1.75, *p* = 0.305)
CVD	1.35 (0.99–1.85, *p* = 0.062)	0.89 (0.61–1.31, *p* = 0.565)
Sex (male)	0.67 (0.45–1.01, *p* = 0.053)	0.66 (0.42–1.03, *p* = 0.069)
PE	1.32 (0.71–2.43, *p* = 0.380)	-
DM	1.20 (0.85–1.70, *p* = 0.302)	-
GERD	0.92 (0.51–1.65, *p* = 0.773)	-
CPFE	1.57 (0.91–2.72, *p* = 0.108)	-
Sleep apnea	1.08 (0.72–1.63, *p* = 0.704)	-
Arrhythmia	1.27 (0.85–1.88, *p* = 0.245)	-
Lung malignancy	1.09 (0.51–2.34, *p* = 0.820)	-
Other malignancies	0.82 (0.51–1.32, *p* = 0.422)	-
Depression or Anxiety	0.67 (0.35–1.29, *p* = 0.226)	-
Neurological ischemic disorders	1.27 (0.74–2.17, *p* = 0.379)	-
Thyroid disease	1.07 (0.73–1.57, *p* = 0.722)	-
Dyslipidemia	1.04 (0.70–1.54, *p* = 0.849)	-
Smoking group 1. Current smoker	-	-
2. Former smoker	1.88 (0.69–5.11, *p* = 0.215)	-
3. Never smoked	2.07 (0.74–5.82, *p* = 0.167)	-
Familial IPF	0.91 (0.57–1.46, *p* = 0.708)	-
Antifibrotic treatment	0.68 (0.44–1.06, *p* = 0.090)	-

Abbreviations: AH—Arterial Hypertension; BMI—Body Mass Index; CPFE—Combined Pulmonary Fibrosis and Emphysema; CVD—Cardiovascular Diseases; DM—Diabetes mellitus; FVC% pred.—Forced Vital Capacity as a Percentage of Predicted Value; GERD—Gastroesophageal Reflux Disease; IPF—Idiopathic Pulmonary Fibrosis; PH—Pulmonary Hypertension; PE—Pulmonary Embolism.

**Table 4:** Definition of survival nodes in recursive partitioning analysis.

Node	Definition	n (%)
2	Patients with FVC < 50% predicted	89 (27.5)
4	Patients with FVC ≥ 50% predicted and no PH	185 (57.1)
5	Patients with FVC ≥ 50% predicted and PH present	50 (15.4)

Abbreviations: FVC% pred.—Forced Vital Capacity as a Percentage of Predicted Value; PH—Pulmonary Hypertension.

**Table 5:** Hazard ratios (HR) for survival by node.

Node	n (%)	HR (Univariable)	HR (Multivariable)
2	89 (27.5)	-	-
4	185 (57.1)	0.29 (0.20–0.41, *p* < 0.001)	0.29 (0.20–0.41, *p* < 0.001)
5	50 (15.4)	0.88 (0.59–1.33, *p* = 0.557)	0.88 (0.59–1.33, *p* = 0.557)

Abbreviations: HR—Hazard Ratio.

## Data Availability

Data supporting reported results can be found at eurILDreg—Portal für Medizinische Datenmodelle (MDM-Portal).
